# Spatial Patterns of Planktonic Fungi Indicate Their Potential Contributions to Biological Carbon Pump and Organic Matter Remineralization in the Water Column of South China Sea

**DOI:** 10.3390/jof9060640

**Published:** 2023-06-01

**Authors:** Kalyani Sen, Mohan Bai, Jiaqian Li, Xueyan Ding, Biswarup Sen, Guangyi Wang

**Affiliations:** 1 Centre for Marine Environmental Ecology, School of Environmental Science and Engineering, Tianjin University, Tianjin 300072, China; ksen@tju.edu.cn (K.S.); bmh@zju.edu.cn (M.B.); lijiaqian@tju.edu.cn (J.L.); 2022214045@tju.edu.cn (X.D.); bsen@tju.edu.cn (B.S.); 2 Key Laboratory of Systems Bioengineering (Ministry of Education), Tianjin University, Tianjin 300072, China; 3 Center for Biosafety Research and Strategy, Tianjin University, Tianjin 300072, China

**Keywords:** mycoplankton, bacterioplankton, abundance, diversity, trophic modes, metabolic function

## Abstract

Fungi have long been known to be dynamic in coastal water columns with multiple trophic modes. However, little is known about their interactions with abiotic and biotic components, contribution to the biological carbon pump (BCP), and organic matter remineralization in the oceanic water column. In this study, we investigated how fungi vary spatially and how their variations relate to that of bacteria in the water column of the South China Sea (SCS). Fungi were about three orders less prevalent than bacteria, and the main factors influencing their distribution were depth, temperature, and distance from the sites of riverine inputs. The decline in the abundance of fungi with depth was less steep than that of bacteria. Correlation tests revealed a strong positive association between the abundance of fungi and bacteria, especially in the twilight (r = 0.62) and aphotic (r = 0.70) zones. However, the co-occurrence network revealed mutual exclusion between certain members of fungi and bacteria. The majority of fungi in the water column were saprotrophs, which indicated that they were generally involved in the degradation of organic matter, particularly in twilight and aphotic zones. Similar to bacteria, the involvement of fungi in the metabolism of carbohydrates, proteins, and lipids was predicted, pointing to their participation in the turnover of organic carbon and the biogeochemical cycling of carbon, nitrogen, and sulfur. These findings suggest that fungi play a role in BCP and support their inclusion in marine microbial ecosystem models.

## 1. Introduction

Mycoplankton, also referred to as planktonic fungi, has caught the attention of researchers globally, and there have been several studies on their abundance, diversity, and putative roles in coastal environments [1,2,3,4,5,6]. However, there are only a handful of studies on the mycoplankton in oceanic waters [7,8,9], and therefore much more information remains unknown. Nevertheless, previous studies have revealed spatiotemporal variations in the community structure and the impact of environmental factors, thereby providing indirect evidence of their roles in the coastal and oceanic water columns [10]. However, sporadic reports do not provide a comprehensive understanding of the contribution of mycoplankton to the marine microbial food web.

As fungi are usually armed with a myriad of extracellular enzymes, they are speculated to convert particulate organic matter (POM) to dissolved organic matter (DOM) [11,12], utilize DOM [12,13], help in the macro aggregation of POM [14], and contribute to the biological carbon pump (BCP) [15,16]. Despite the increasing evidence for their multifaceted roles [1,4,11,17], marine ecosystem models have yet to consider fungi as an important heterotrophic component similar to bacteria [18]. Over the past decade, there has been some progress in understanding the relationship between fungi and other planktonic members of the marine ecosystem, e.g., bacterioplankton, phytoplankton, and zooplankton [1,2,6,11,19]. These studies have revealed that fungal biomass declines with depth and is often similar to that of prokaryotes or correlates with the biomass of phytoplankton. Thus far, only bacteria have been considered the principal heterotrophs in marine ecosystems in terms of biomass [15]. Our current understanding of fungal biomass as a heterotrophic component, especially in oceanic water columns, remains poor. Thus, more holistic efforts are required to uncover the nature of the fungi–bacteria relationship, integrate fungi into the marine food web, and redraw the marine ecosystem model by filling in gaps and joining the missing links.

The South China Sea (SCS) is a marginal tropical sea with a maximum depth of about 5000 m and a complex pattern of ocean currents, upwelling, eddies, and gyres. SCS experiences a monsoon system with a reversal of wind and current direction in the summer and winter months [20]. Although the bacterial community structure of the SCS water column has been described to some extent [21,22], studies on the contrasting community structures of mycoplankton and bacterioplankton and their possible interactions in the SCS water column are missing. In this study, we elucidate the depth structuring of the abundance and diversity of mycoplankton and bacterioplankton in the SCS, their interactions, and their putative contribution to organic matter remineralization. This study provides the first lines of evidence suggesting the significant contribution of mycoplankton, similar to that of bacterioplankton, to BCP and organic matter remineralization.

## 2. Materials and Methods

### 2.1. Collection of Water Samples

**Figure 1:**
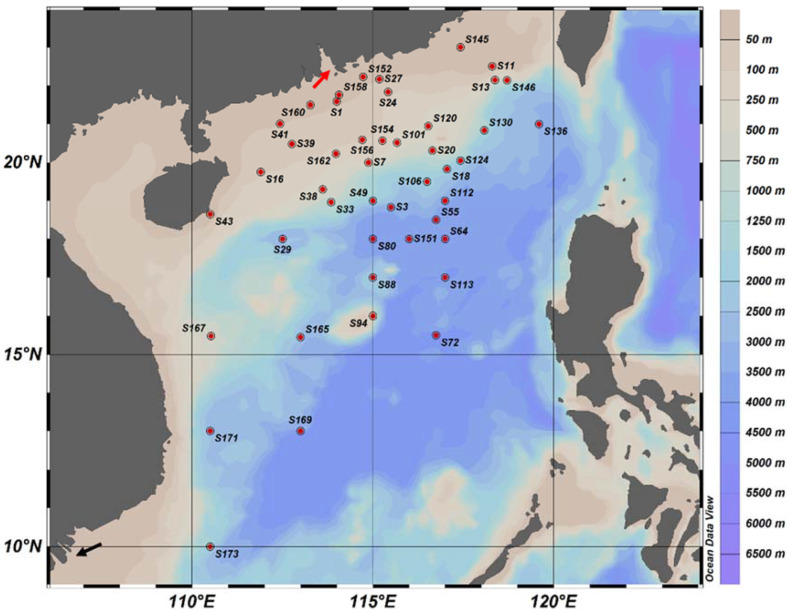
Map of sampling stations in the South China Sea. Only the surface stations are shown. The red and black arrows indicate the mouths of the Pearl River and Mekong River, respectively.

Seawater samples were collected from various depths using Sea-Bird CTD rosette samplers during three independent cruises to the South China Sea in the summer months of 2016 to 2018 (Figure 1). A total of 182 water samples, ranging from the surface (5 m) to a maximum depth of 4000 m, were processed (Table S1). The water subsample (2 L) was filtered through a 0.22 μm polycarbonate filter membrane (Millipore, Burlington, MA, USA) for DNA extraction. The resulting filters were stored at −80 °C until further processing. The environmental parameters, including temperature, salinity, and chlorophyll a, were measured using CTD samplers. The water samples were stored at 4 °C in sterile plastic bottles and analyzed in the laboratory for the concentration of silicate, ammonium, total phosphate, and nitrite by following the standard protocols as mentioned in “Specifications for oceanographic survey-Part 4: Survey of chemical parameters in seawater (GB/T 12763.4-2007)”. The distance from the mouth of the Pearl River was calculated using the coordinates 113.57° E and 22.29° N as the reference, while for the Mekong River, the reference coordinates were 106.41° E, 10.47° N. The “*distm*” function of the R package *Geosphere* was used to calculate the distance of each station from the respective reference coordinates. The samples were classified according to their zones of sampling, namely euphotic (epipelagic), twilight (mesopelagic), and aphotic (bathypelagic). The vertical zonation of the water column into euphotic (<200 m), twilight (200–800 m), and aphotic (≥1000 m) zones was based on the literature [23,24].

### 2.2. Fungal and Bacterial Community Analysis

#### 2.2.1. DNA Extraction

The E.Z.N.A.^TM^ Water DNA Kit (Omega Bio-tek, Norcross, GA, USA) was used to extract DNA from filters stored at −80 °C. The extracted DNA was suspended in 100 μL sterile water and held at −20 °C until further processing. A total of 50 filters were chosen for further community analysis. While all these filters were processed for fungal community analysis, 43 filters were processed for bacterial community analysis.

#### 2.2.2. PCR Amplification

Fungal ITS amplicons were generated using the primer pairs ITS1-F/ITS2 (ITS1-F: 5′CTTGGTCATTTAGAGGAAGTAA3′ and ITS2: 5′ GCTGCGTTCTTCATCGATGC3′) [25,26]. PCR amplification was performed using barcoded primer pairs. The PCR mixture (25 µL) contained 2X Taq Plus Master Mix (12.5 µL), 1 µL of each primer (5 µM), 30 ng (X µL) template DNA, and (7.5−X µL) H_2_O. Thermocycling consisted of an initial denaturation at 94 °C for 5 min, followed by 32 cycles of 94 °C for 30 s, 55 °C for 30 s, 72 °C for 60 s, and a final extraction at 72 °C for 7 min. Three separate reactions were conducted to account for potentially heterogeneous amplification from the environmental template for each sample. The PCR products were purified using the AXYGEN Gel Extraction Kit (QIAGEN, Hilden, Germany) and quantified using quantitative PCR (qPCR). An equimolar mix of all three amplicon libraries was used for sequencing. The amplicons were paired-end sequenced on an Illumina platform by commercial sequencing facilities (Allwegene Ltd., Beijing, China, and Biomarker Technology Corporation, Beijing, China).

Bacterial 16S rRNA amplicons were generated using the primer pairs 338F (5′-ACTCCTACGGGAGGCAGCA-3′) and 806R (5′-GGACTACHVGGGTWTCTAAT-3′) that target the V3–V4 region of 16S rRNA [27]. The amplicons were paired-end sequenced on an Illumina platform by Biomarker Technology Corporation (Beijing, China). Thermocycling consisted of an initial denaturation at 98 °C for 2 min, followed by 30 cycles of 98 °C for 30 s, 50 °C for 30 s, 72 °C for 60 s, and a final extraction at 72 °C for 5 min.

#### 2.2.3. Downstream Processing of Sequencing Reads

The raw reads were demultiplexed and assigned to the respective samples according to their barcodes. The demultiplexed clean reads (barcodes and adapters removed) were imported into QIIME 2^TM^ (2018.8) (https://qiime2.org/). All downstream analysis was carried out on the QIIME 2 platform using only the forward reads of each primer pair. The noisy sequences were filtered, errors were corrected, and chimeric sequences and singletons were removed and dereplicated using the DADA2 method to generate the amplicon sequence variants (ASVs) [28]. The single-end reads were truncated at 240 bases based on sequence quality. The ASV tables generated by ITS and 16S rRNA markers were used to assign taxonomies. Taxonomy was assigned to the fungal ASVs using a consensus blast method of the “feature-classifier” plugin in QIIME 2^TM^ [29] against the full UNITE+INSD dataset (version 18.11.2018, https://doi.org/10.15156/BIO/786349) with a similarity threshold of 0.95 and coverage of 0.5. The ASVs that could not be classified using UNITE were classified using the “BROCC” plugin in QIIME 2^TM^ against the NCBI’s nucleotide database [30]. The classified ASVs were further filtered to retain those that belonged to the kingdom of Fungi. From 50 samples, 9035 ASVs were generated, of which 3154 were fungal ASVs.

For bacteria, taxonomy was assigned using the consensus blast method of the “feature-classifier” plugin in QIIME 2^TM^ [29] against the SILVA database (version 132, https://www.arb-silva.de/no_cache/download/archive/qiime/) with a similarity threshold of 0.95 and coverage of 0.5. The classified ASVs were further filtered to retain those that belonged to the kingdom of Bacteria. From 43 samples, 10,333 ASVs were generated, of which 9193 were bacterial ASVs.

### 2.3. Quantitative PCR Analysis

#### 2.3.1. Fungal 18S rRNA Gene Copies

Quantitative PCR using the fungi-specific 18S rRNA gene was performed using the method described by Taylor and Cunliffe [5]. Briefly, a 10 μL reaction mixture contained 5 μL of 2X ChamQ Vazyme SYBR qPCR master mix, 0.4 μL of each primer (0.4 μΜ), 1 μL of DNA template, and 3.2 μL nuclease-free molecular-grade water. A CFX Connect^TM^ Real-Time System (Bio-Rad CFX Connect Real-Time System) was used to perform qPCR. The following qPCR regime was used: denaturation at 94 °C for 3 min, 40 cycles at 94 °C for 10 s, annealing at 50 °C for 15 s, and elongation and acquisition of fluorescence data at 72 °C for 20 s. A standard curve was constructed using known amounts of target templates generated by PCR amplification of the target gene from the genomic DNA of *Rhodosporidium* sp. TJUWZ4 (CGMCC #2.5689, GenBank accession: KT281890.1), as previously described [31].

#### 2.3.2. Bacterial 16S rRNA Gene Copies

Quantitative PCR using the bacteria-specific 16S rRNA gene was performed using the method described elsewhere [27]. The 16S rRNA gene primers (338F: 5′-ACTCCTRCGGGAGGCAGCAG-3′, 806R: 5′-GGACTACCVGGGTATCTAAT-3′) were used to amplify the 16S rRNA gene in the extracted DNA. A 10 μL reaction mixture contained 5 μL of 2X ChamQ Vazyme SYBR qPCR master mix, 0.4 μL of each primer (0.4 μΜ), 1 μL of DNA template, and 3.2 μL nuclease-free molecular-grade water. A CFX Connect^TM^ Real-Time System (Bio-Rad CFX Connect Real-Time System) was used to perform qPCR. The following qPCR regime was used: denaturation at 94 °C for 3 min, 40 cycles at 94 °C for 15 s, annealing at 50 °C for 30 s, and elongation and acquisition of fluorescence data at 72 °C for 30 s. A standard curve was constructed using known amounts of target template generated by PCR amplification of the target gene from genomic DNA of *E. coli,* as previously described (see Section 2.3.1).

### 2.4. Statistical Analysis

The Kruskal–Wallis test of significance and the Dunn test were used to determine whether there were statistically significant differences between groups and subgroups, respectively. Correlations between abundance and environmental variables were determined using Pearson’s correlation test after log transformation of the abundance data. Spearman’s rank correlation test was used to calculate the correlation between mycoplankton and bacterioplankton abundances for different subgroups. Principal component analysis (PCA) was performed to analyze the gradients of the environmental variables. All statistical analyses and data plotting were done in R software (v3.3.1).

### 2.5. Prediction of Trophic Modes

The trophic modes of fungal ASVs were determined using FUNGuild [32], a bioinformatic tool involving a two-component system. The FUNGuild tool includes online community annotated databases and a Python script that assigns functional information to ASVs. The tool searches taxonomic strings in the ASV table against the online Guilds database (Fungi), which contains taxonomic keywords and functional metadata. The output of this tool is the original ASV table with functional metadata appended (https://github.com/UMNFuN/FUNGuild). For guild assignments, only fungal ASVs matching a UNITE reference sequence with ≥95% sequence similarity were retained and used as the input of this tool. Furthermore, to avoid overinterpretation of the data ecologically, only “highly probable” or “probable” guild assignments were accepted and reported.

### 2.6. Network Analysis

A co-occurrence network was constructed with CoNET [33], a plugin in Cytoscape software, following the method described elsewhere [34]. We employed an ensemble approach combining four different measures: two measures of dissimilarity (Bray–Curtis (BC) and Kullback–Leibler (KLD)) and two measures of correlation (Pearson and Spearman correlation). The dataset of 43 samples used in the network analysis contained fungal and bacterial ASVs in rows and samples in columns. The rows were divided by their sums before the computation of BC and KLD measures. The row minimum occurrence parameter was set at 25. To test the statistical significance of the edge scores, we computed measure- and edge-specific permutation and bootstrap score distributions with 1000 iterations each. Using a pooled variance, the *p* values were then computed by *z*-scoring the permuted null and bootstrap confidence intervals. Edges with scores not within the 95% confidence interval of the bootstrap distribution were removed.

### 2.7. Identification of Metabolic Functions

The functional profiles of the communities were identified using the PICRUSt2 tool [35]. This tool involves a series of steps, including phylogenetic placement, hidden-state prediction, and sample-wise gene abundance tabulation. The PICRUSt2 pipeline inputs amplicon sequencing data and outputs stratified gene family and metabolic pathway abundances. In this study, we used fungal and bacterial ASV tables and their DNA sequences generated in the sequence processing steps described in Section 2.2.3. The output of PICRUSt2 was visualized and statistically analyzed using the STAMP v2.1.3 tool [36].

## 3. Results

### 3.1. Environmental Characteristics in Relation to Various Water Zones

The waters in the euphotic, twilight, and aphotic zones had significantly different temperatures, salinities, and levels of chlorophyll *a* (Table S2). High chlorophyll levels were found near the SCS’s coastal areas, and rainfall was observed over the SCS during the summer, according to satellite images from Terra/MODIS (Figure S1) and Aqua/MODIS (Figure S2). Additionally, the ecological characteristics of the euphotic zone were different from those of the twilight and aphotic zones, according to the PCA of the environmental parameters and nutrient data (Figure S3). PC1 and PC2 explained 72.5% of the total variation in the environmental data.

### 3.2. Distribution and Abundance Patterns

**Figure 2:**
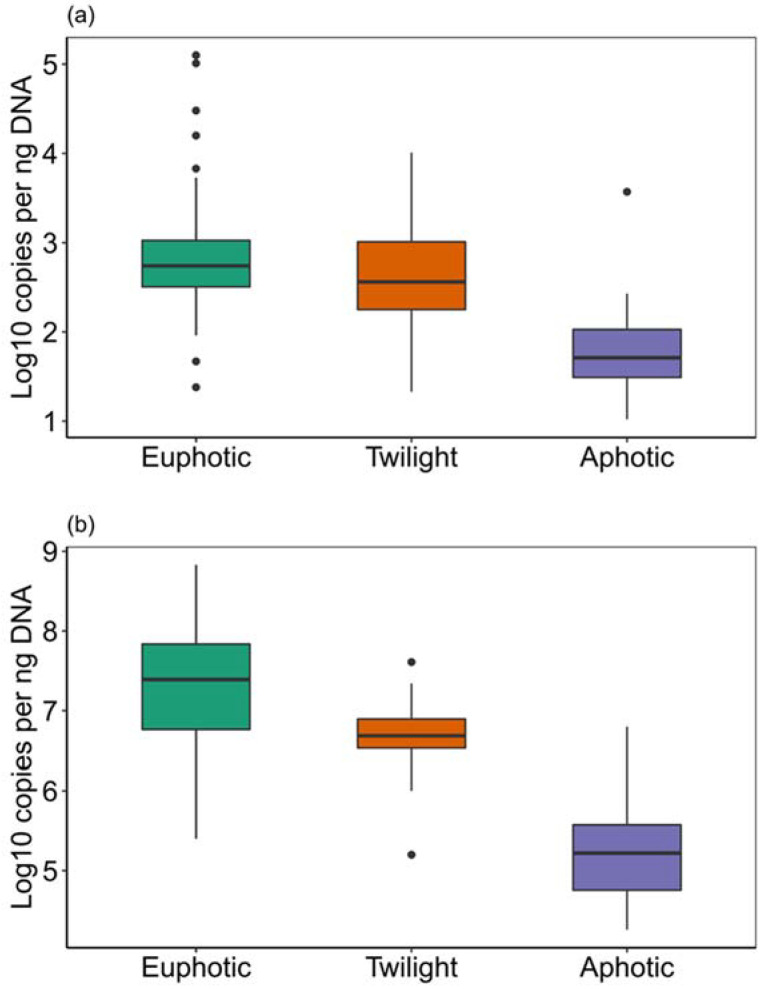
Box plots showing depth-related changes in the abundances of (**a**) fungi and (**b**) bacteria.

Overall, mycoplankton abundance ranged from 10 to 10^5^ 18S rRNA gene copies/ng DNA (Table 1, Figure 2a). Their abundance varied significantly (*p* < 0.05; ANOVA) between the euphotic, twilight, and aphotic zones, with the aphotic zone having the lowest. The abundance showed a weakly negative correlation (*r* = −0.28) with salinity and a strong positive correlation (*r* = 0.49) with temperature, according to Pearson’s correlation test. The relationship between abundance and depth was found to be inverse (*r* = −0.50). A weak positive correlation (*r* = 0.13) was found between abundance and the distance from the mouth of the Mekong River, but a weak negative correlation (*r* = −0.22) was found with the distance from the mouth of the Pearl River. Because riverine inputs are known to contain fungi in the form of spores and tissues [3,37,38], it is very likely that fungi are brought in by the Pearl River into SCS. As a result, with the increase in distance from Pearl River, the abundance gradually decreased. The main effect (*p* < 0.05, ANOVA) was determined to be the distance from the Pearl River, and the interaction effect between the distances from the Pearl and Mekong rivers was also significant (*p* < 0.05, ANOVA).

In comparison to mycoplankton, the abundance of bacterioplankton was about three orders of magnitude higher, ranging from 10^4^ to 10^8^ gene copies/ng DNA. Additionally, there were significant differences in bacterioplankton abundances (*p* < 0.05; ANOVA) across the depth zones (Table 1, Figure 2b). The euphotic and twilight zones had comparable abundances, whereas the aphotic zone had the lowest abundance of all the zones (*p* > 0.05, ANOVA). Notably, bacterioplankton was more abundant in the aphotic zone than mycoplankton by several orders of magnitude. The abundance of bacterioplankton showed a trend that was similar to the trend seen for mycoplankton, showing a negative correlation with depth (*r* = −0.55) and salinity (*r* = −0.38) but a positive correlation (*r* = 0.62) with temperature. The abundance of bacterioplankton also showed a weakly positive correlation with the distance from the mouth of the Mekong River (*r* = 0.14) but a weakly negative correlation with the distance from the mouth of the Pearl River (*r* = −0.33).

### 3.3. Variations in Community Composition Related to Various Water Zones

**Figure 3:**
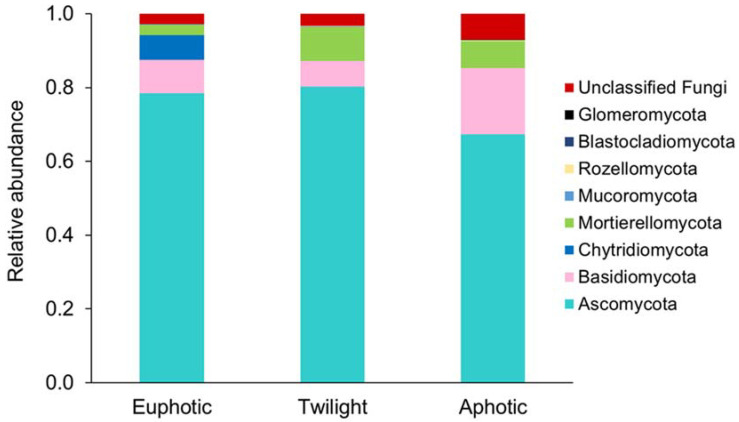
Composition of fungal communities in various water zones at the phylum level.

The environmental DNA isolated from 50 representative SCS water samples was subjected to high-throughput sequence analysis, which revealed a total of 10 phyla, 39 classes, and 352 genera of fungi. The different taxa recovered, in order of their relative abundance, were Ascomycota (78.8%, 13 classes), Basidiomycota (8.3%, 11 classes), Mortierellomycota (6.9%, one class), unclassified Fungi (3.3%), Chytridiomycota (2.4%, four classes), Rozellomycota (0.12%, one class), Glomeromycota (0.06%, two classes), Mucoromycota (0.05%, four classes), Blastocladiomycota (0.04%, one class), Zoopagomycota (0.0004%, one class), and Olpidiomycota (0.0003%, one class). The proportions of Ascomycota in the euphotic (78.51%) and twilight (80.20%) zones were relatively higher than those in the aphotic zone (67.27%) (Figure 3). Sordariomycetes (42.4%, 56.49%) and Saccharomycetes (4.67%, 3.32%) were the most prevalent classes of Ascomycota in the euphotic and twilight zones, respectively, while Dothideomycetes (28.03%) dominated the aphotic zone (Figure S4). Similar to Ascomycota, Chytridiomycota (6.78%, 0.06%, and 0.07%) showed a declining trend. In contrast, the proportions of Basidiomycota were lower in the upper zones (8.96%, 6.95%) compared to the aphotic zone (17.96%), similar to Mortierellomycota (2.81%, 9.26%, 7.22%). The classes of Basidiomycota that were most abundant in the aphotic zone were Tremellomycetes (12.46%), Agaricomycetes (1.94%), and Malasseziomycetes (1.12%).

Further investigation of the various zones’ taxonomic compositions of the two communities, namely mycoplankton and bacterioplankton, was conducted. When compared to the aphotic zone, the proportion of each dominant bacterial phylum was relatively higher in the upper zones (euphotic and twilight) (Table S3). This was once more in contrast to the results of the fungal pattern, which showed that the proportions of a few phyla, such as Basidiomycota and Mortierellomycota, were relatively lower in the upper zone.

### 3.4. Interactions between Planktonic Communities

**Figure 4:**
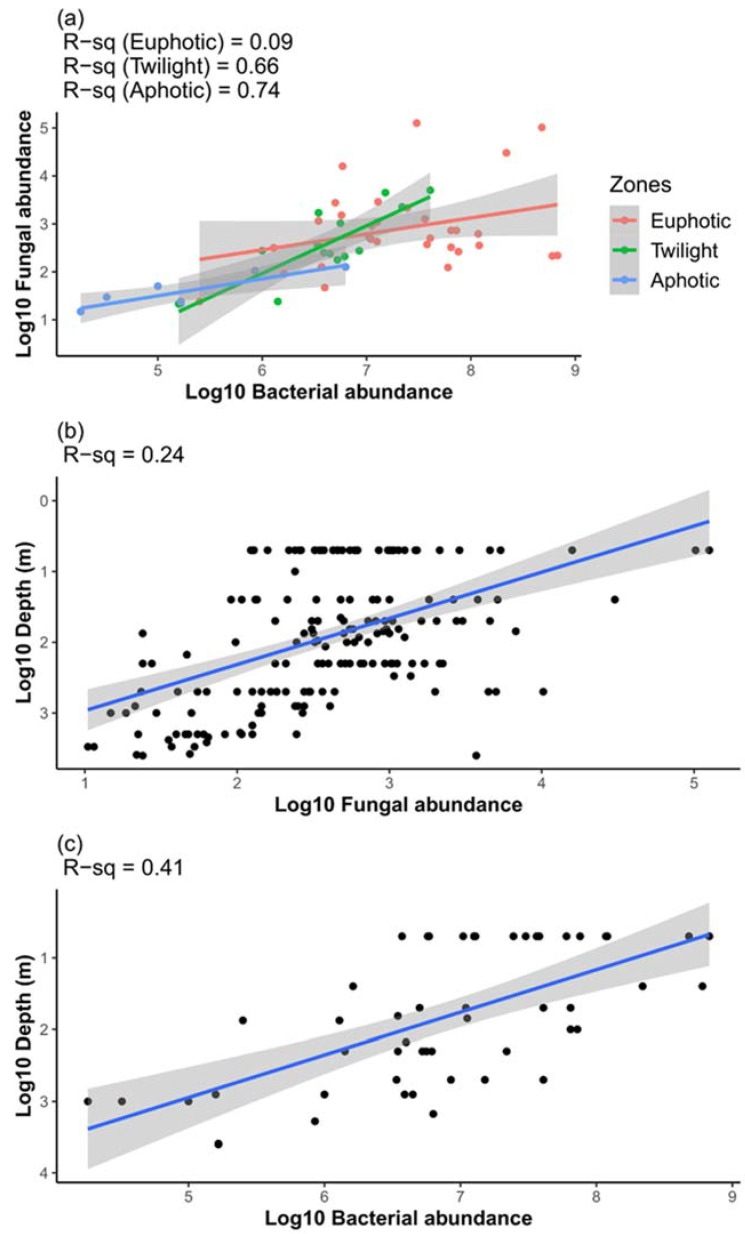
Log-log plots of (**a**) fungal abundance vs. bacterial abundance, (**b**) fungal abundance vs. depth, and (**c**) bacterial abundance vs. depth.

The abundance of mycoplankton and bacterioplankton showed an increasing strength of positive association from the top to the bottom of the water column (Figure 4a). Additionally, to understand how mycoplankton and bacterioplankton abundances change with depth, the logarithmic abundance was regressed with the logarithmic depth, and the resulting slope was determined. The abundance of bacterioplankton exhibited a steeper slope (−0.69) (Figure 4c) than that of mycoplankton (slope = −0.36) (Figure 4b).

**Figure 5:**
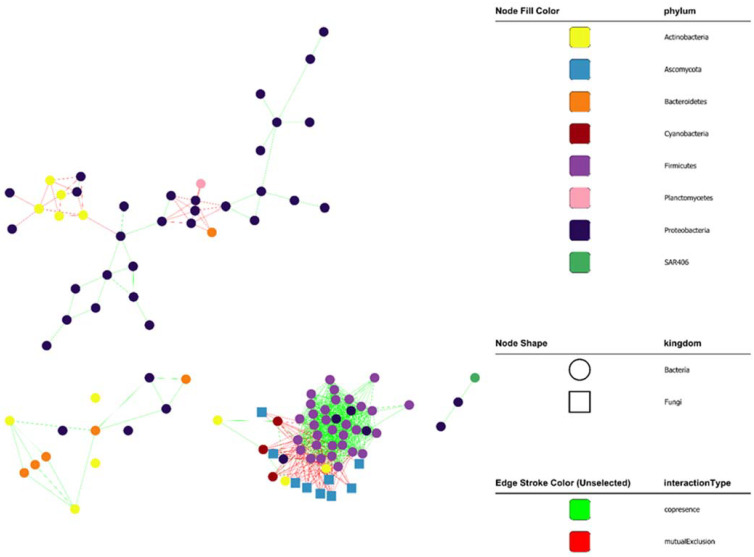
Cooccurrence network of fungi and bacteria.

To further understand the interactions between the two plankton communities, a co-occurrence network was constructed and evaluated. The resulting network had 104 nodes and a density of 0.125 with four connected components and 12.82 average number of neighbors (Figure 5). The co-occurrence network produced five clusters, one of which was large and contained both bacteria and fungi, while the other four clusters contained only bacteria. The largest cluster (cluster #1) had 47 nodes (vertices) and 582 edges (links) and contained both bacteria and fungi. At the phylum level, the members of cluster #1 were Firmicutes (31 nodes), Ascomycota (8 nodes), Cyanobacteria (three nodes), Proteobacteria (three nodes), and Actinobacteria (two nodes). Cluster #2 had eight nodes and 13 edges with only Proteobacteria. Cluster #3 had five nodes and seven edges with Actinobacteria (three nodes) and Proteobacteria (two nodes). Cluster #4 had four nodes and five edges with a-Proteobacteria (three nodes) and g-Proteobacteria (one node). Cluster #5 had four nodes and five edges with Bacteroidetes (two nodes) and Proteobacteria (two nodes). Cluster #1 consisted of unclassified genera (24 nodes), two fungal genera: *Cladosporium* (two nodes) and *Alternaria* (one node), and seven bacterial genera: *Lactococcus* (five nodes), *Streptococcus* (three nodes), *Alkaliphilus* (two nodes), *Leuconostoc* (two nodes), *Synechococcus* (two nodes), *Bacillus* (two nodes), *Carnobacterium* (two nodes), and *Enterococcus* (two nodes). Bacterioplankton were numerous, closely clustered, and had positive edges in cluster #1 while mycoplankton were few and primarily had negative edges. *Cladosporium–Lactococcus*, *Cladosporium–Carnobacterium*, *Cladosporium–Enterococcus*, and *Alternaria–Lactococcus* were found to be mutually exclusive. Interestingly, all of these bacteria belong to the lactic acid bacteria (LAB) group and are known to show antifungal activity.

### 3.5. Trophic Modes of Mycoplankton

To determine the trophic modes of the mycoplankton communities of SCS, the FUNGuild tool was employed in this study. Based on the FUNGuild analysis output, the classified genera were majorly grouped into six trophic modes: saprotroph, pathotroph, symbiotroph, pathotroph–symbiotroph, pathotroph–saprotroph, and saprotroph–symbiotroph (Table 2). Among these trophic modes, saprotroph (46.01%), pathotroph (32.46%), and pathotroph–saprotroph (15.46%) were comparatively the most dominant in the water column of SCS. Notably, the abundance of saprotroph mode in the euphotic zone was significantly lower than in the twilight and aphotic zones. Furthermore, the abundance of fungi with dual trophic modes, i.e., pathotroph–symbiotroph, was considerably higher in the twilight zone compared with that of the other two zones, a finding similar to that of saprotrophic fungi.

### 3.6. Metabolic Potential of Plankton Communities

**Figure 6:**
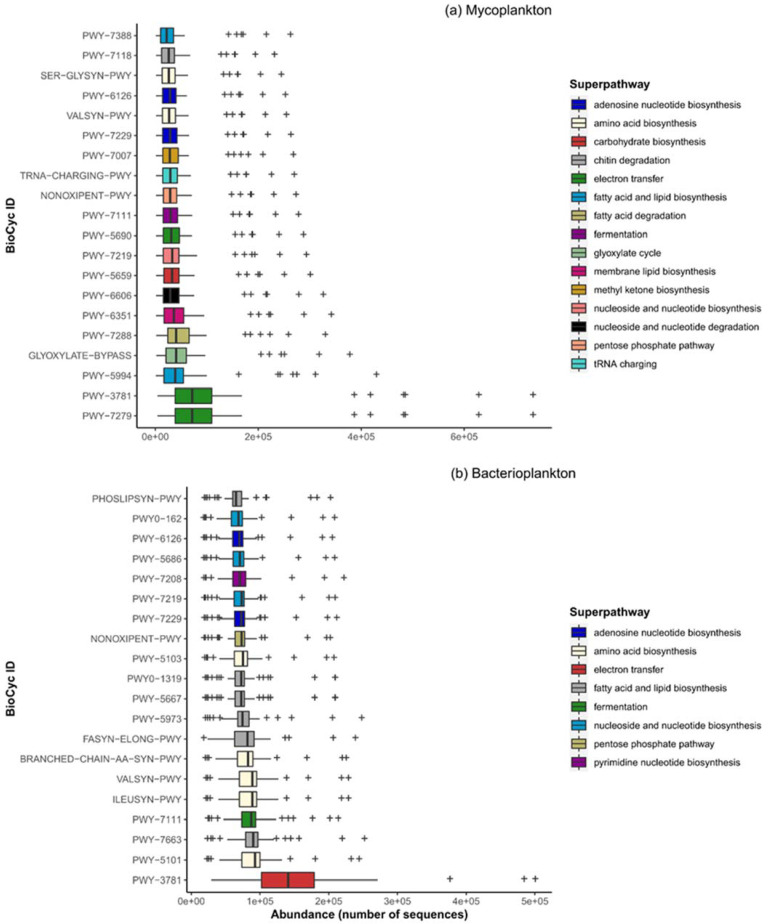
Predicted top 20 abundant metabolic pathways in the metagenomes of (**a**) fungi and (**b**) bacteria. The functional predictions were made using the PICRUSt2 tool.

To elucidate the metabolic potential of the mycoplankton and bacterioplankton communities in SCS, metagenomes were predicted using the PICRUSt2 bioinformatic tool. Protein-encoding genes involved in 75 different pathways that perform the broad function of biosynthesis, degradation/utilization/assimilation, or generation of precursor metabolites and energy were predicted in the mycoplankton metagenomes of marine environments. The top 10 (abundant) pathways across all samples were aerobic respiration I and II (electron transport), fatty acid and lipid biosynthesis, glyoxylate cycle, fatty acid beta oxidation, membrane lipid biosynthesis, nucleoside and nucleotide degradation, carbohydrate biosynthesis, and nucleoside and nucleotide biosynthesis (Figure 6a). The other functions included metabolism of amino acids, carbohydrates and energy, fatty acids and lipids, glycogen biosynthesis, nucleotides, nitrogen, sulfur, and other compounds, such as vitamins, octane, methyl ketone, heme, secondary metabolites, amine and polyamine biosynthesis, which may be the main metabolic processes in marine mycoplankton.

In contrast to mycoplankton, protein-encoding genes involved in 434 different pathways within the classes, namely biosynthesis, degradation/utilization/assimilation, or generation of precursor metabolite and energy, were predicted for bacterioplankton metagenomes. The top 10 pathways were for aerobic respiration, amino acid biosynthesis, fatty acid and lipid biosynthesis, and fermentation (Figure 6b). Other less abundant pathways included fatty acid and lipid degradation, nucleoside and nucleotide biosynthesis, secondary metabolite biosynthesis, cell structure biosynthesis, co-factor, carrier, vitamin biosynthesis, etc. Some degradative pathways that were common between the metagenomes of the two communities were fatty acid degradation, urea cycle, chitin degradation, galactose degradation, L-tyrosine degradation, sulfate reduction, sucrose degradation, L-leucine and L-tryptophan degradation, and octane oxidation.

## 4. Discussion

### 4.1. Fungal Abundance Patterns and Their Significance in the Water Column

Quantifying fungal abundance is essential for understanding their roles in the marine water column. Fungi were found to be distributed throughout the SCS water column from the surface to the deep waters. It has been previously reported that fungal abundance decreases with the depth of the water column and is mainly associated with phytoplankton abundance patterns [11,12,16]. This possibly explains why the abundance was higher in the euphotic zone than in the other zones. Furthermore, fungal abundance has been reported to be high in marine snow [16] and is also associated with sinking algae [39]. Thus, the presence of fungi in the entire water column could be attributed to their association with sinking particulate matter, such as marine snow or algal detritus. Compared to the aphotic zone, the twilight zone exhibited higher abundance than the euphotic zone. This can result from the twilight zone’s biogeochemical features, including receiving organic matter from the euphotic zone, creating hotspots with enhanced biodiversity, and conditions ideal for fungal colonization and survival [23,24]. In addition, the twilight zone hosts diverse forms of life, including fish, crustaceans, jellies, worms, and squids. Fungi that are pathogens and parasites of such marine organisms are likely to be found in that zone [40,41]. Organic matter-rich animal excreta, sinking in fecal pellets from surface waters, are also known to be colonized by fungi in the twilight zone [12]. Being mainly aerobic organisms, fungi might fail to grow in deep waters, leading to their low abundance in the aphotic zone. A decreased abundance of bacteria with depth has also been reported previously [11,23]. Overall, the findings of this study suggest depth as a significant factor that determines the differences in the mycoplankton abundances of SCS.

### 4.2. Abiotic and Biotic Interactions and Their Implications

Fungi exhibited a significant negative correlation with the abiotic factors, namely depth, salinity, and temperature of the water column, a trend similar to that exhibited by the bacteria. A previous study on the coastal waters of station L4, Plymouth also reported a negative correlation of abundance with salinity and suggested transportation of fungi to coastal water by riverine inflow [5]. Fungal biomass in water columns has been reported to generally decline with a decrease in temperature [3]. A reduction in the abundance of bacteria with depth has also been reported earlier [11,23]. Thus, both microbial communities respond similarly to the abiotic factors.

In this study, the biotic interactions between fungi and bacteria were determined based on the correlation analysis of their abundance along the depth of the water column. Interestingly, our study provides strong evidence of syntrophic interactions between the two microbial communities, specifically in the twilight and aphotic waters of the SCS. The syntrophic interaction between fungi and bacteria seems beneficial, especially in deep waters, possibly due to the labile resource limitation. Thus, recalcitrant organic matter in deep waters can be efficiently utilized if fungi and bacteria are in a syntrophic relationship. If such a relationship exists in the water column, then it will impact the BCP.

Oceanic BCP is vital in exporting organic matter produced in the euphotic zone to the twilight and aphotic zones, thereby sequestering carbon in the deep ocean [42]. The heterotrophic communities can potentially alter the functioning of BCP either negatively or positively [43]. The ocean’s deep waters receive particulate and dissolved organic matter from the surface, which is subject to microbial mineralization in the water column [44]. A significant portion of this sinking organic matter is recalcitrant and resistant to mineralization by bacteria [45]. This recalcitrant organic matter is generally considered sequestered in the deep ocean, as fungi have not been considered in any marine ecological model. Fungi are known to be better degraders of recalcitrant organic matter than bacteria. Thus, bacteria can benefit from the breakdown products of fungi in deep waters.

Moreover, the combined activity of fungi and bacteria would result in a highly efficient micro-bioreactor leading to the mineralization of sinking organic matter in the ocean [3,11]. Recent studies have reported that both microbial communities are able to uptake algal polysaccharides in vitro [1]. However, the nature of the interactions between these communities remained unresolved. Fungi can uptake the recalcitrant organic matter—the phytoplankton detritus and exudates—because they secrete hydrolytic enzymes that break down the complex and larger organic molecules [11]. In contrast, bacteria, owing to their small size and high-affinity transport systems with saturation constants of up to 10^−8^ M, are more efficient in the uptake of labile organic matter or DOM [44,46]. Previous research has found that the presence of labile litter increases bacterial biomass, whereas the presence of recalcitrant litter increases fungal abundance [47], supporting the different capacities of the two communities in the uptake of labile and recalcitrant organic matter.

Bacterial abundance exhibited a steeper slope than fungal abundance (Figure 4b,c), suggesting a sharp decrease with depth. Similar slope values ranging from −0.55 to −1.0 have been previously reported for bacteria [23]. However, to date, no reports have been available on the slope of the relationship between mycoplankton abundance and depth. Furthermore, the present study revealed that the decline in mycoplankton abundance is gradual but relatively sharp for bacterioplankton abundance. Compared to mycoplankton (R^2^ = 0.24), the bacterioplankton abundance relationship with depth was stronger (R^2^ = 0.41). A possible explanation for the gradual decrease in mycoplankton but a more substantial decrease in bacterioplankton with depth could be the tolerance of fungi to low temperature and high hydrostatic pressure and their ability to utilize a wide range of substrates [16]. Fungi have been reported to have the potential to form and stabilize macroaggregates and to help in carbon sequestration [11,14,16]. However, our study reveals that fungi, known to exhibit wide biodegradative potential together with the ability to survive in extreme environments, might strongly influence recalcitrant organic matter degradation and POM-DOM cycling in the deep sea. It may thus have dual roles in promoting and reversing BCP.

Although it is known that ascomycetous fungi dominate marine waters [12,48], the present study further revealed their depth-related variations. Remarkably, the increased proportion of Dothideomycetes in the deep waters evident in the present study agreed with earlier reports [48]. Furthermore, the pattern observed for Basidiomycota in this study has been reported earlier [49,50]. The yeast forms of Basidiomycota are known to dominate deep-sea environments, and they are not constrained by the need for osmotrophy [51]. These results showed that the proportions of Ascomycota and Chytridiomycota generally decreased with the depth of the water column, whereas those of Basidiomycota and Mortierellomycota increased. In contrast, the proportions of all the dominant bacterial phyla, namely Proteobacteria, Cyanobacteria, Firmicutes, Actinobacteria, and Bacteroidetes, decreased with the depth of the water column (Table S3). Overall, the comparative analysis revealed that although fungi and bacteria are heterotrophic, the depth-related patterns of their dominant phyla can differ considerably.

Previous studies on the interactions between fungi and bacteria in freshwater ecosystems have suggested antagonistic and synergistic interactions between them [52,53,54]. However, only a few studies have speculated about the potential interactions between fungi and bacteria in marine environments [1,2,11]. Those studies were limited to coastal environments and did not provide evidence of the possible interactions between fungi and bacteria in the oceanic environment. A previous study speculated that there might be competition and/or syntrophic relationships between fungi and bacteria in the marine environment [1]. Interestingly, our study revealed mutual exclusion among the members of the two microbial communities of the SCS (Figure 5). One possible explanation for this mutual exclusion could be the competition for similar resources. For example, mycoplankton and bacterioplankton have both been reported to utilize algal polysaccharides [1]. Utilizing algal polysaccharides suggests that *Cladosporium* is an active saprotroph in marine environments [1]. Therefore, the presence of *Cladosporium* and *Alternaria* in the cooccurrence network indicated their importance in the water column and the possible utilization of algal detritus. Furthermore, the cooccurrence network revealed the presence of mutual exclusion, such as the interaction between *Cladosporium*-*Lactococcus*, *Cladosporium*-*Carnobacterium*, *Cladosporium*-*Enterococcus*, and *Alternaria*-*Lactococcus*. The mechanism underlying the mutual exclusion between these members of fungi and bacteria in the water column was the antifungal activity of lactic acid bacteria (LAB), namely *Lactococcus*, *Carnobacterium*, and *Enterococcus*. The antifungal activity of LAB has been previously reported for the prevention of food spoilage [55,56]. These findings suggest that LAB might be instrumental in controlling the fungal community and preventing fungal metabolism in macro aggregates, such as the marine snow, which would ultimately aid in stabilizing aggregates, promoting sinking, and leading to carbon sequestration in the water column.

### 4.3. Implications of Trophic Modes and Metabolic Functions of Mycoplankton

Earlier studies have speculated that fungi can affect algal bloom dynamics by playing multiple roles, including saprotroph, pathogen, or symbiont [57]. Additionally, saprotrophic fungi have been previously reported to be abundant in marine environments [48,58,59]. On comparison of the various depth zones, this study revealed that the abundance of saprotroph modes of fungi in the euphotic zone was significantly lower than that of the twilight and aphotic zones (Table 2). The dominance of saprotrophic fungi in the twilight zone was not surprising because this zone is a hot spot for marine life and microbial activity [23,24]. Moreover, the availability of sinking organic matter from the euphotic zone and marine animal carcasses makes it ideal for the activity of saprotrophic fungi. Thus, saprotrophic fungi in the twilight and aphotic zones have the potential to alter the efficiency of the BCP and might affect carbon cycling in the ocean and accelerate global warming. Interestingly, studies have indicated that fungal abundance in oceanic waters might increase under acidic conditions resulting from global warming [60]. Therefore, it is imperative to address fungi that were previously underestimated and ignored in models of marine ecosystems.

A previous study reported decreased saprotrophic fungal OTUs (operational taxonomic units) from surface to deep waters [48]. However, another study found saprotrophic fungi to be most abundant in deep marine sediments [59]. Fungal distribution in deep waters has been noted to be patchy, with pockets of high fungal populations [61]. It may thus be speculated that the dominance of saprotrophic fungi observed in the aphotic zone could result from resuspension from sediments or sinking from the twilight zone, along with organic matter. Furthermore, the abundance of fungi with dual trophic modes in the twilight zone was significantly higher than that in the other zones (Table 2). The presence of fungi with dual trophic modes suggests an adaptive survival strategy in conditions with resource limitations. Overall, this study revealed that saprotroph and pathotroph, the most dominant trophic modes of fungi in the water column of SCS, exhibited depth-related differences in their abundance. Additionally, mycoplankton with dual trophic modes indicated an adaptive strategy for better survival under nutrient limitations.

The water column has several energy resources, such as marine snow, sinking organic matter, cellular and structural components of dead organisms, and live organisms that serve as hosts. A better understanding of the activities of mycoplankton in the water column would help define their role in biogeochemical cycling. Based on metatranscriptomics data, a recent study reported on the mechanisms utilized by fungi to cope with seasonal environmental stresses in coastal ecosystems [62]. Fungi have been found to be metabolically active in the marine sediments [63], Munida Microbial Observatory Time-Series (MOTS) transect across the subtropical frontal zone in the Pacific east of New Zealand, and TARA Oceans project at the surface and different depths [8], but our study provides the first lines of evidence for metabolically active mycoplankton in the SCS water column similar to that of bacterioplankton. Furthermore, we report the involvement of mycoplankton in carbohydrate, protein, and lipid metabolism in the water column of SCS, suggesting its role in organic carbon turnover. The presence of carbohydrate degradation pathways, especially that for chitin, suggests that mycoplankton might be feeding on this abundant polymer usually derived from the dead remains of crustaceans and zooplankton. The existence of polysaccharides and protein degradation capabilities in mycoplankton has also been recently reported [64,65,66,67]. In addition, we report the presence of vitamin synthesis pathways within the mycoplankton metagenome. This supports an earlier report [8] that suggested fungi as a source of vitamins, especially in deeper waters where there are no phytoplankton. Furthermore, L-amino acids have been reported to be released from phytoplankton through grazing [23], and the detection of L-amino acid degradation pathways suggests the breakdown of phytoplanktonic detritus by the mycoplankton community. Overall, the metabolic potential of mycoplankton suggests their involvement in ocean biogeochemical cycling similar to bacterioplankton.

## Figures and Tables

**Table 1:** Average mycoplankton and bacterioplankton abundances in various oceanic zones.

Zones	Mycoplankton (18S rRNA Gene Copies/Ng DNA)	Bacterioplankton (16S rRNA Gene Copies/Ng DNA)
Euphotic	3588 ± 1585 ^b^	(9.09 ± 3.1) × 10^7 b^
Twilight	923 ± 241 ^c^	(8.67 ± 2.95) × 10^6 a^
Aphotic	194 ± 122 ^a^	(1.09 ± 0.87) × 10^6 a^

Note: Groups sharing a letter not significantly different (*alpha* = 0.05); values are mean ± SE; ANOVA by Kruskal–Wallis rank sum test, post hoc test by Dunn test (*p* value adjusted with the Benjamini–Hochberg method).

**Table 2:** Spatial variations of major trophic modes of mycoplankton communities in the SCS.

Trophic Modes	Euphotic	Twilight	Aphotic
Pathotroph	1471 ± 518 ^a^	3040 ± 934 ^a^	811 ± 248 ^a^
Saprotroph	1003 ± 263 ^b^	6116 ± 1675 ^a^	2038 ± 535 ^a^
Symbiotroph	80.7 ± 25.6 ^a^	335 ± 131 ^a^	162 ± 84.5 ^a^
Pathotroph–Saprotroph	699 ± 179 ^a^	1419 ± 372 ^a^	514 ± 149 ^a^
Pathotroph–Symbiotroph	14.4 ± 9.36 ^a^	348 ± 145 ^b^	32.8 ± 22.9 ^ab^
Saprotroph–Symbiotroph	18.5 ± 8.24 ^a^	23.2 ± 17.5 ^a^	11.0 ± 9.11 ^a^

Note: The values represent the number of sequences, which are based on rarefied data (rarefied to minimum sampling depth). Groups sharing a letter are not significantly different (*alpha* = 0.05); values are mean ± SE; ANOVA by Kruskal-Wallis test and post hoc test by Dunn test (*p* value adjusted with Benjamini–Hochberg method).

## Data Availability

The data have been deposited with links to BioProject accession no. PRJNA602152 and PRJNA602157 in the NCBI BioProject database (https://www.ncbi.nlm.nih.gov/bioproject/). Data may be available from the corresponding author upon reasonable request.

## Supplementary Materials

The following supporting information can be downloaded at: https://www.mdpi.com/article/10.3390/jof9060640/s1, Figure S1: Satellite images showing temporal variation of chlorophyll concentration along the coastal SCS and progress and intensification of Southwest Monsoon over SCS during the study period (May 2016–September 2018). The images indicate the presence of a Typhoon (Mangkhut) on 15 September 2018. Terra/MODIS images generated from https://worldview.earthdata.nasa.gov/ (accessed on 20 December 2018); Figure S2: Satellite images showing temporal variation of chlorophyll concentration in the waters of the South China Sea during the sampling period (May 2016–September 2018). Aqua/MODIS images were generated and analyzed online at https://neo.sci.gsfc.nasa.gov/analysis/; Figure S3: PCA illustrating the variations in the environmental characteristics of various water zones; Figure S4: Variations in the taxonomic composition of mycoplankton communities between different water zones at the class level; Table S1: Metadata of samples analyzed in the present study; Table S2: Environmental parameters and nutrient concentrations of water samples from different depth zones; Table S3: Proportions of the most dominant (relative abundance ≥ 1%) bacterial phyla in various water zones.
