# Supplementary material for: Spatial Patterns of Planktonic Fungi Indicate Their Potential Contributions to Biological Carbon Pump and Organic Matter Remineralization in the Water Column of South China Sea
Source: J Fungi (Basel). 2023 Jun 1;9(6):640. doi: 10.3390/jof9060640 (PMC10304542; doi:10.3390/jof9060640)
Supplement: Supplementary file 1 [file jof-09-00640-s001.zip › jof-2387025-supplementary.pdf]

Supplementary Information

Spatial Patterns of Planktonic Fungi Indicate Their Potential Contributions to Biological Carbon Pump and Organic Matter Remineralization in the Water Column of South China Sea

Kalyani Sen [1], Mohan Bai [1], Jiaqian Li [1], Xueyan Ding [1], Biswarup Sen [1],

and Guangyi Wang[1,2,3,*]

[1] Centre for Marine Environmental Ecology, School of Environmental Science and Engineering, Tianjin University, Tianjin 300072, China

[2] Key Laboratory of Systems Bioengineering (Ministry of Education), Tianjin University, Tianjin 300072, China

[3] Center for Biosafety Research and Strategy, Tianjin University, Tianjin 300072, China

* Corresponding author: gywang@tju.edu.cn

Table S1: Metadata of samples analyzed in the present study.

Sample	Station	Sampling Date	Long. (°E)	Lat. (°N)	Depth (m)	Temp. (°C)	Salinity (psu)	Chlorophyll a (mg/m^3)	Total Phosphate (mmol/L)	Nitrite (mmol/L)	Ammonium (mmol/L)	Silicate (mmol/L)
S1	A1	2016.05	114	21.58	5	26.784	34.169	0	n.a	n.a	n.a	n.a
S10	A6	2016.05	114.88	20	500	8.713	34.404	0	n.a	n.a	n.a	n.a
S100	A4	2017.07	115	16	3000	2.35	34.64	0	n.a	n.a	n.a	n.a
S101	B1	2017.07	115.67	20.52	5	29.37	34.18	0	n.a	n.a	n.a	n.a
S102	B1	2017.07	115.67	20.52	25	28.85	34.33	0	n.a	n.a	n.a	n.a
S103	B1	2017.07	115.67	20.52	65	22.04	34.74	0.95	n.a	n.a	n.a	n.a
S104	B1	2017.07	115.67	20.52	200	13.88	34.6	0	n.a	n.a	n.a	n.a
S105	B1	2017.07	115.67	20.52	4000	2.43	34.63	0	n.a	n.a	n.a	n.a
S106	B3	2017.07	116.5	19.5	5	29.46	33.79	0	n.a	n.a	n.a	n.a
S107	B3	2017.07	116.5	19.5	25	29.34	33.78	0	n.a	n.a	n.a	n.a
S108	B3	2017.07	116.5	19.5	65	22.77	34.6	0.88	n.a	n.a	n.a	n.a
S109	B3	2017.07	116.5	19.5	500	7.95	34.47	0	n.a	n.a	n.a	n.a
S11	D1	2016.05	118.3	22.5	5	28.49	34.513		n.a	n.a	n.a	n.a
S110	B3	2017.07	116.5	19.5	1000	4.34	34.6	0	n.a	n.a	n.a	n.a
S111	B3	2017.07	116.5	19.5	2000	2.44	34.65	0	n.a	n.a	n.a	n.a
S112	B4	2017.07	117	19	5	29.73	33.86	0	n.a	n.a	n.a	n.a
S113	BL-12	2017.07	117	17	5	29.09	33.54	0	n.a	n.a	n.a	n.a

S114	BL-12	2017.07	117	17	25	28.9	33.55	0	n.a	n.a	n.a	n.a
S115	BL-12	2017.07	117	17	75	22.15	34.7	0.52	n.a	n.a	n.a	n.a
S116	BL-12	2017.07	117	17	200	14.3	34.63	0	n.a	n.a	n.a	n.a
S117	BL-12	2017.07	117	17	500	8.43	34.51	0	n.a	n.a	n.a	n.a
S118	BL-12	2017.07	117	17	2000	2.52	34.62	0	n.a	n.a	n.a	n.a
S119	BL-12	2017.07	117	17	2600	2.37	34.63	0	n.a	n.a	n.a	n.a
S12	D1	2016.05	118.3	22.5	50	24.385	34.877		n.a	n.a	n.a	n.a
S120	C1	2017.07	116.53	20.95	5	28.91	34.07	0.05	n.a	n.a	n.a	n.a
S121	C1	2017.07	116.53	20.95	50	25.49	34.48	0.99	n.a	n.a	n.a	n.a
S122	C1	2017.07	116.53	20.95	200	14.53	34.61	0	n.a	n.a	n.a	n.a
S123	C1	2017.07	116.53	20.95	300	11.3	34.49	0	n.a	n.a	n.a	n.a
S124	C3	2017.07	117.42	20.05	5	30.19	33.87	0	n.a	n.a	n.a	n.a
S125	C3	2017.07	117.42	20.05	25	29.94	33.91	0	n.a	n.a	n.a	n.a
S126	C3	2017.07	117.42	20.05	65	22.76	34.66	0.83	n.a	n.a	n.a	n.a
S127	C3	2017.07	117.42	20.05	200	14.17	34.6	0	n.a	n.a	n.a	n.a
S128	C3	2017.07	117.42	20.05	500	8.5	34.48	0	n.a	n.a	n.a	n.a
S129	C3	2017.07	117.42	20.05	2000	2.4	34.63	0	n.a	n.a	n.a	n.a
S13	D4	2016.05	118.38	22.14	5	28.312	34.546		n.a	n.a	n.a	n.a
S130	D4	2017.07	118.08	20.84	5	29.73	33.87	0	n.a	n.a	n.a	n.a
S131	D4	2017.07	118.08	20.84	25	29.7	33.9	0	n.a	n.a	n.a	n.a
S132	D4	2017.07	118.08	20.84	65	24.77	34.91	0.7	n.a	n.a	n.a	n.a

S133	D4	2017.07	118.08	20.84	200	14.8	34.66	0	n.a	n.a	n.a	n.a
S134	D4	2017.07	118.08	20.84	500	8.83	34.48	0	n.a	n.a	n.a	n.a
S135	D4	2017.07	118.08	20.84	1900	2.54	34.64	0	n.a	n.a	n.a	n.a
S136	E1	2017.06	119.6	21	5	29.95	33.89	0	n.a	n.a	n.a	n.a
S137	E1	2017.06	119.6	21	25	29.95	33.89	0	n.a	n.a	n.a	n.a
S138	E1	2017.06	119.6	21	115	24.71	34.75	0.35	n.a	n.a	n.a	n.a
S139	E1	2017.06	119.6	21	200	16.95	34.75	0	n.a	n.a	n.a	n.a
S14	D4	2016.05	118.38	22.14	50	26.185	34.707		n.a	n.a	n.a	n.a
S140	E1	2017.06	119.6	21	300	12.09	34.46	0	n.a	n.a	n.a	n.a
S141	E1	2017.06	119.6	21	500	8.25	34.41	0	n.a	n.a	n.a	n.a
S142	E1	2017.06	119.6	21	1000	4.29	34.56	0	n.a	n.a	n.a	n.a
S143	E1	2017.06	119.6	21	2000	2.38	34.63	0	n.a	n.a	n.a	n.a
S144	E1	2017.06	119.6	21	3000	2.32	34.62	0	n.a	n.a	n.a	n.a
S145	N1	2017.07	117.42	23	5	30.53	34.25	0	n.a	n.a	n.a	n.a
S146	N2	2017.06	118.71	22.13	5	30.53	34.25	0	n.a	n.a	n.a	n.a
S147	N2	2017.06	118.71	22.13	25	30.24	34.39	0	n.a	n.a	n.a	n.a
S148	N2	2017.06	118.71	22.13	100	22.57	34.64	0.93	n.a	n.a	n.a	n.a
S149	N2	2017.06	118.71	22.13	200	17.04	34.67	0	n.a	n.a	n.a	n.a
S15	D4	2016.05	118.38	22.14	150	18.517	34.776		n.a	n.a	n.a	n.a
S150	N2	2017.06	118.71	22.13	500	8.11	34.47	0	n.a	n.a	n.a	n.a
S151	SEATS	2017.07	116	18	5	29.49	33.5	0	n.a	n.a	n.a	n.a

S152	Z01	2018.08	114.73	22.22	5	28.9	31.8	2.16	0.01	0.3	1.18	0
S153	Z01	2018.08	114.73	22.22	25	22.2	34.34	4.86	0.4	0.29	0.41	17.19
S154	Z05	2018.08	115.27	20.57	5	29.43	33.65	0	0.05	0.12	0.48	1.1
S155	Z05	2018.08	115.27	20.57	100	18.78	34.62	0	n.a	n.a	n.a	n.a
S156	Z10	2018.08	114.71	20.59	5	28.98	33.63	0	0	0.11	0.56	1.64
S157	Z10	2018.08	114.71	20.59	50	26.56	34.08	0.01	0	0.11	0.43	2.86
S158	Z14	2018.08	114.06	21.75	5	28.69	32.39	4.03	0	0.22	0.43	0.91
S159	Z14	2018.08	114.06	21.75	25	26.5	33.9	0.19	0	0.08	0.38	0
S16	E6	2016.06	111.9	19.75	5	28.88	34.212	0	n.a	n.a	n.a	n.a
S160	Z15	2018.08	113.27	21.49	5	28.62	33.11	19.72	0	0.26	0.45	3.11
S162	Z19	2018.08	113.98	20.23	5	29.06	33.7	0	0	0.16	0.73	3.69
S163	Z19	2018.08	113.98	20.23	50	25.07	34.21	0.37	0.14	0.07	0.49	7.06
S164	Z19	2018.08	113.98	20.23	100	20.03	34.6	0	n.a	n.a	n.a	n.a
S165	Z46	2018.09	113	15.45	5	29.22	33.78	0	0.07	0.06	0.05	0
S166	Z46	2018.09	113	15.45	1000	4.48	34.5	0	2.26	0.34	0.39	130.85
S167	Z51	2018.09	110.522	15.479	5	26.86	34.49	0	0	0	0.35	9.66
S168	Z51	2018.09	110.522	15.479	500	8.24	34.72	0	1.4	0	0.6	65.61
S169	Z58	2018.09	113	13	5	27.55	33.37	0.09	0	0.01	0.37	0.11
S17	E6	2016.06	111.9	19.75	100	21.161	34.717	0.24	n.a	n.a	n.a	n.a
S170	Z58	2018.09	113	13	1000	4.5	34.45	0	1.05	0.04	0.34	115.98
S171	Z63	2018.09	110.498	13	5	27.93	34.01	0	0.01	0.03	0.54	0

S172	Z63	2018.09	110.498	13	1000	4.33	34.88	0	2.43	0.29	0.73	131.58
S173	Z76	2018.09	110.5	10	5	28.66	32.98	0.12	0.1	0.08	0.43	1.55
S174	Z76	2018.09	110.5	10	1000	4.42	34.45	0	1.74	0.04	0.26	19.81
S175	Z05	2018.09	115.27	20.57	50	23.17	34.43	0.68	0.16	0.29	0.36	0.04
S176	Z10	2018.09	114.71	20.59	100	19.71	34.61	0	n.a	n.a	n.a	n.a
S177	Z58	2018.09	113	13	200	14.61	34.46	0	1.11	0	0.36	30.24
S178	Z58	2018.09	113	13	500	8.94	34.35	0	0.82	0.02	0	38.9
S179	Z63	2018.09	110.5	13	25	27.44	34.06		0.01	0.03	0.54	0
S18	F1	2016.05	117.05	19.83	5	29.289	34.582	0	n.a	n.a	n.a	n.a
S180	Z63	2018.09	110.5	13	500	8.403	34.75	0	2.4	0	0.4	85.53
S181	Z76	2018.09	110.5	10	200	16.26	34.51	0	n.a	n.a	n.a	n.a
S182	Z76	2018.09	110.5	10	500	8.9437	34.3631	0	n.a	n.a	n.a	n.a
S183	Z39	2018.09	110.8	17.6	1000	4.41	34.61	0	n.a	n.a	n.a	n.a
S19	F1	2016.05	117.05	19.83	100	18.487	34.713	0.11	n.a	n.a	n.a	n.a
S2	A1	2016.05	114	21.58	45	21.426	34.537	4.33	n.a	n.a	n.a	n.a
S20	F3	2016.05	116.65	20.31	5	28.916	34.542	0	n.a	n.a	n.a	n.a
S21	F3	2016.05	116.65	20.31	75	22.432	34.901	0.12	n.a	n.a	n.a	n.a
S22	F3	2016.05	116.65	20.31	200	16.525	34.646	0	n.a	n.a	n.a	n.a
S23	F3	2016.05	116.65	20.31	500	8.538	34.418	0	n.a	n.a	n.a	n.a
S24	F8	2016.05	115.42	21.83	5	28.764	34.211	0	n.a	n.a	n.a	n.a
S25	F8	2016.05	115.42	21.83	50	21.088	34.664	0.13	n.a	n.a	n.a	n.a

S26	F8	2016.05	115.42	21.83	85	20.876	34.695	0.13	n.a	n.a	n.a	n.a
S27	F9	2016.05	115.18	22.16	5	28.615	31.72	0	n.a	n.a	n.a	n.a
S28	F9	2016.05	115.18	22.16	50	21.49	34.693	0.16	n.a	n.a	n.a	n.a
S29	K7	2016.06	112.5	18	5	30.5	34.11	0	n.a	n.a	n.a	n.a
S3	A10	2016.05	115.5	18.83	5	29.938	33.779	0	n.a	n.a	n.a	n.a
S30	K7	2016.06	112.5	18	50	19.963	34.683	0.13	n.a	n.a	n.a	n.a
S31	K7	2016.06	112.5	18	200	12.918	34.48	0	n.a	n.a	n.a	n.a
S32	K7	2016.06	112.5	18	1500	2.803	34.599	0	n.a	n.a	n.a	n.a
S33	N1	2016.06	113.85	18.96	5	29.438	34.268	0	n.a	n.a	n.a	n.a
S34	N1	2016.06	113.85	18.96	50	20.013	34.625	0.1	n.a	n.a	n.a	n.a
S35	N1	2016.06	113.85	18.96	75	22.059	34.669	0.11	n.a	n.a	n.a	n.a
S36	N1	2016.06	113.85	18.96	200	15.062	34.56	0	n.a	n.a	n.a	n.a
S37	N1	2016.06	113.85	18.96	800	5.643	34.484	0	n.a	n.a	n.a	n.a
S38	N3	2016.06	113.61	19.3	5	29.671	34.322	0	n.a	n.a	n.a	n.a
S39	N7	2016.06	112.76	20.48	5	29.054	34.152	0	n.a	n.a	n.a	n.a
S4	A10	2016.05	115.5	18.83	50	24.894	34.09	0.1	n.a	n.a	n.a	n.a
S40	N7	2016.06	112.76	20.48	50	23.296	34.693	0.13	n.a	n.a	n.a	n.a
S41	N8	2016.06	112.43	21.01	5	29.078	33.516	0	n.a	n.a	n.a	n.a
S42	N8	2016.06	112.43	21.01	50	21.567	34.578	0.41	n.a	n.a	n.a	n.a
S43	Q7	2016.06	110.5	18.65	5	29.225	33.039	0	n.a	n.a	n.a	n.a
S44	Q7	2016.06	110.5	18.65	50	21.712	34.489	0.3	n.a	n.a	n.a	n.a

S45	SEATS	2016.05	116	18	5	30.086	34.113	0	n.a	n.a	n.a	n.a
S46	SEATS	2016.05	116	18	200	13.959	34.521	0	n.a	n.a	n.a	n.a
S47	SEATS	2016.05	116	18	800	4.457	34.514	0	n.a	n.a	n.a	n.a
S48	SEATS	2016.05	116	18	3000	2.352	34.613	0	n.a	n.a	n.a	n.a
S49	A1	2017.07	115	19	5	29.26	33.92	0	n.a	n.a	n.a	n.a
S5	A10	2016.05	115.5	18.83	500	8.461	34.412	0	n.a	n.a	n.a	n.a
S50	A1	2017.07	115	19	25	28.92	34.05	0	n.a	n.a	n.a	n.a
S51	A1	2017.07	115	19	95	23.59	34.53	0.71	n.a	n.a	n.a	n.a
S52	A1	2017.07	115	19	200	15.87	34.67	0	n.a	n.a	n.a	n.a
S52	Z15	2018.08	113.27	21.49	25	26.54	33.96	0.98	0	0.23	1.12	9.64
S53	A1	2017.07	115	19	500	9.21	34.47	0	n.a	n.a	n.a	n.a
S54	A1	2017.07	115	19	2200	2.37	34.64	0	n.a	n.a	n.a	n.a
S55	A10	2017.07	116.75	18.5	5	29.83	33.64	0	n.a	n.a	n.a	n.a
S56	A10	2017.07	116.75	18.5	25	29.53	33.8	0	n.a	n.a	n.a	n.a
S57	A10	2017.07	116.75	18.5	65	23.1	34.61	0.82	n.a	n.a	n.a	n.a
S58	A10	2017.07	116.75	18.5	200	14.85	34.64	0	n.a	n.a	n.a	n.a
S59	A10	2017.07	116.75	18.5	500	8.17	34.48	0	n.a	n.a	n.a	n.a
S6	A10	2016.05	115.5	18.83	800	5.676	34.467	0	n.a	n.a	n.a	n.a
S60	A10	2017.07	116.75	18.5	800	5.66	34.56	0	n.a	n.a	n.a	n.a
S61	A10	2017.07	116.75	18.5	2000	2.49	34.65	0	n.a	n.a	n.a	n.a
S62	A10	2017.07	116.75	18.5	3000	2.35	34.63	0	n.a	n.a	n.a	n.a

S63	A10	2017.07	116.75	18.5	3800	2.38	34.63	0	n.a	n.a	n.a	n.a
S64	A11	2017.07	117	18	5	29.67	33.76	0	n.a	n.a	n.a	n.a
S65	A11	2017.07	117	18	25	29.59	33.76	0	n.a	n.a	n.a	n.a
S66	A11	2017.07	117	18	75	21.97	34.65	0.73	n.a	n.a	n.a	n.a
S67	A11	2017.07	117	18	200	14.66	34.63	0	n.a	n.a	n.a	n.a
S68	A11	2017.07	117	18	500	8.59	34.49	0	n.a	n.a	n.a	n.a
S69	A11	2017.07	117	18	800	5.8	34.54	0	n.a	n.a	n.a	n.a
S7	A6	2016.05	114.88	20	5	29.162	34.059	0	n.a	n.a	n.a	n.a
S70	A11	2017.07	117	18	2000	2.5	34.64	0	n.a	n.a	n.a	n.a
S71	A11	2017.07	117	18	3900	2.39	34.63	0	n.a	n.a	n.a	n.a
S72	A14	2017.07	116.75	15.5	5	28.47	33.42	0	n.a	n.a	n.a	n.a
S73	A14	2017.07	116.75	15.5	25	28.46	33.43	0	n.a	n.a	n.a	n.a
S74	A14	2017.07	116.75	15.5	75	24.05	34.47	0.43	n.a	n.a	n.a	n.a
S75	A14	2017.07	116.75	15.5	200	15.52	34.64	0	n.a	n.a	n.a	n.a
S76	A14	2017.07	116.75	15.5	500	8.84	34.49	0	n.a	n.a	n.a	n.a
S77	A14	2017.07	116.75	15.5	800	5.7	34.57	0	n.a	n.a	n.a	n.a
S78	A14	2017.07	116.75	15.5	2000	2.48	34.64	0	n.a	n.a	n.a	n.a
S79	A14	2017.07	116.75	15.5	4000	2.43	34.63	0	n.a	n.a	n.a	n.a
S8	A6	2016.05	114.88	20	70	21.684	34.648	0.13	n.a	n.a	n.a	n.a
S80	A2	2017.07	115	18	5	27.34	34.16	0	n.a	n.a	n.a	n.a
S81	A2	2017.07	115	18	25	26.92	34.31	0	n.a	n.a	n.a	n.a

S82	A2	2017.07	115	18	85	23.99	34.78	0.47	n.a	n.a	n.a	n.a
S83	A2	2017.07	115	18	200	17.48	34.73	0	n.a	n.a	n.a	n.a
S84	A2	2017.07	115	18	500	9.11	34.45	0	n.a	n.a	n.a	n.a
S85	A2	2017.07	115	18	1000	4.69	34.56	0	n.a	n.a	n.a	n.a
S86	A2	2017.07	115	18	2000	2.51	34.63	0	n.a	n.a	n.a	n.a
S87	A2	2017.07	115	18	2400	2.39	34.63	0	n.a	n.a	n.a	n.a
S88	A3	2017.07	115	17	10	27.8	33.76	0	n.a	n.a	n.a	n.a
S89	A3	2017.07	115	17	25	27.84	33.76	0	n.a	n.a	n.a	n.a
S9	A6	2016.05	114.88	20	200	14.028	34.502	0	n.a	n.a	n.a	n.a
S90	A3	2017.07	115	17	70	22.98	34.57	0.6	n.a	n.a	n.a	n.a
S91	A3	2017.07	115	17	200	14.85	34.61	0	n.a	n.a	n.a	n.a
S92	A3	2017.07	115	17	500	8.61	34.47	0	n.a	n.a	n.a	n.a
S93	A3	2017.07	115	17	2000	2.47	34.65	0	n.a	n.a	n.a	n.a
S94	A4	2017.07	115	16	5	28.91	33.62	0	n.a	n.a	n.a	n.a
S95	A4	2017.07	115	16	25	28.83	33.61	0	n.a	n.a	n.a	n.a
S96	A4	2017.07	115	16	75	22.47	34.68	0.68	n.a	n.a	n.a	n.a
S97	A4	2017.07	115	16	200	14.07	34.59	0	n.a	n.a	n.a	n.a
S98	A4	2017.07	115	16	500	8.31	34.48	0	n.a	n.a	n.a	n.a
S99	A4	2017.07	115	16	2000	2.54	34.64	0	n.a	n.a	n.a	n.a

Table S2 Environmental parameters and nutrient concentrations of water samples from different depth zones.

**Parameters/nutrients**	**Euphotic**	**Twilight**	**Aphotic**
Depth (m)	33.7 ± 3.32 ^{b}	400 ± 29.3 ^{c}	2177 ± 172 ^{a}
Temperature (°C)	26.4 ± 0.35 ^{b}	11.1 ± 0.54 ^{c}	2.91 ± 0.16 ^{a}
Salinity (psu)	34.5 ± 0.06 ^{b}	34.1 ± 0.01 ^{c}	34.6 ± 0.01 ^{a}
Chlorophyll *a* (mg/m^3)	0.52±0.22	ND	ND
Total Phosphate (PO_4^{3-}) (mmol/L)	0.053 ± 0.024 ^{b}	1.43 ± 0.344 ^{a}	1.87 ± 0.31 ^{a}
Nitrite (mmol/L)	0.136 ± 0.024 ^{a}	0.005 ± 0.005 ^{b}	0.178 ± 0.08 ^{a}
Ammonium (NH_4^+) (mmol/L)	0.517 ± 0.062 ^{a}	0.34 ± 0.125 ^{a}	0.43 ± 0.104 ^{a}
Silicate (SiO_2) (mmol/L)	3.25 ± 1.11 ^{b}	55.1 ± 12.6 ^{a}	99.6 ± 26.8 ^{a}

Note: Groups sharing a letter are not significantly different (alpha = 0.05); values are mean ± SE; ND = below detection level; ANOVA by Kruskal Wallis rank sum test, post hoc test by Dunn test (*p* value adjusted with Benjamini-Hochberg method.

Table S3 Proportions of the most dominant (relative abundance ≥ 1%) bacterial phyla in various water zones

**Phyla**	**Euphotic**	**Twilight**	**Aphotic**
Proteobacteria	61.2	31.5	7.29
Cyanobacteria	73.9	23	3.06
Firmicutes	46.3	38.6	15
Actinobacteria	50	45.2	4.77
Bacteroidetes	68.7	28.9	2.45
Marinimicrobia	40.4	48.7	11
Chloroflexi	16.6	67.6	15.8

Note: values are percentage relative abundance.

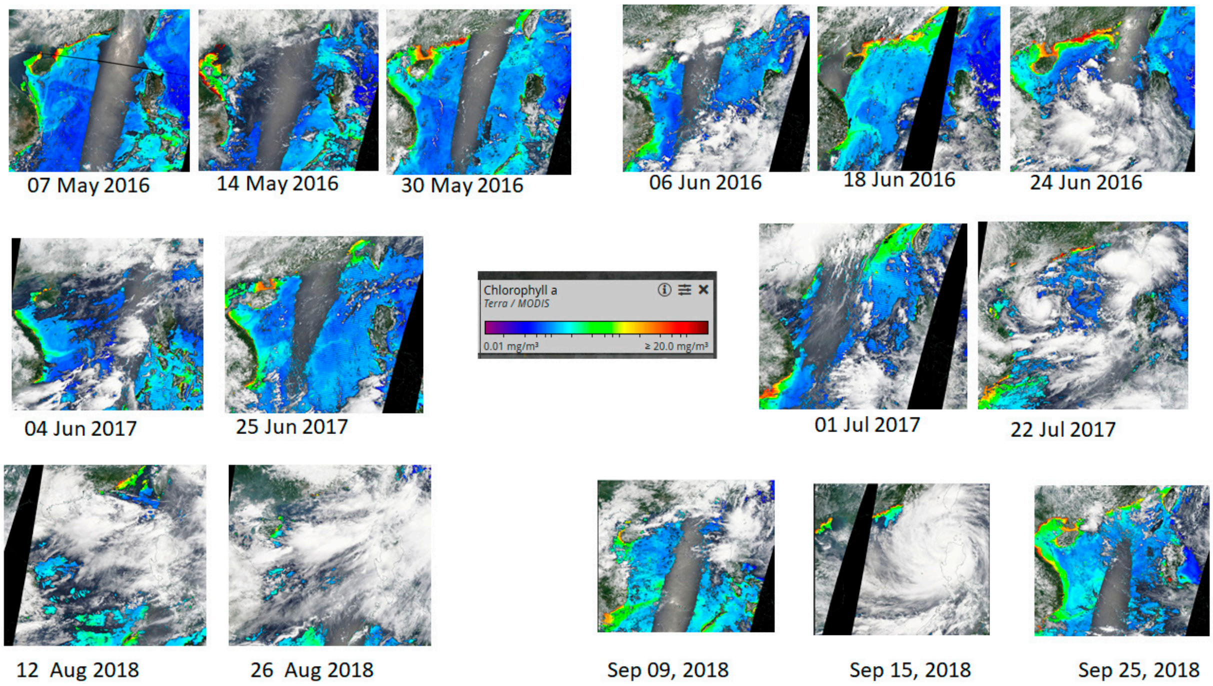

Figure S1. Satellite images showing temporal variation of chlorophyll concentration along the coastal SCS and progress and intensification of Southwest Monsoon over SCS during the study period (May 2016–September 2018). The images indicate the presence of a Typhoon (Mangkhut) on September 15, 2018. Terra/MODIS images generated from https://worldview.earthdata.nasa.gov/.

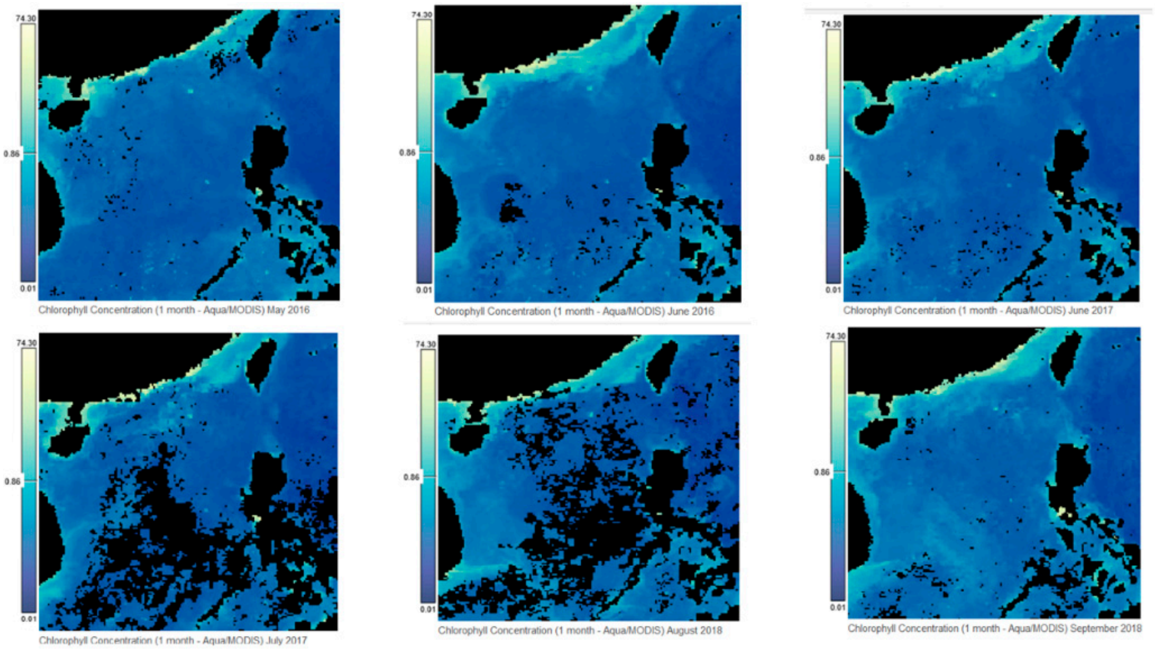

Figure S2. Satellite images showing temporal variation of chlorophyll concentration in the waters of South China Sea during the sampling period (May 2016–September 2018). Aqua/MODIS images were generated and analyzed online at https://neo.sci.gsfc.nasa.gov/analysis/.

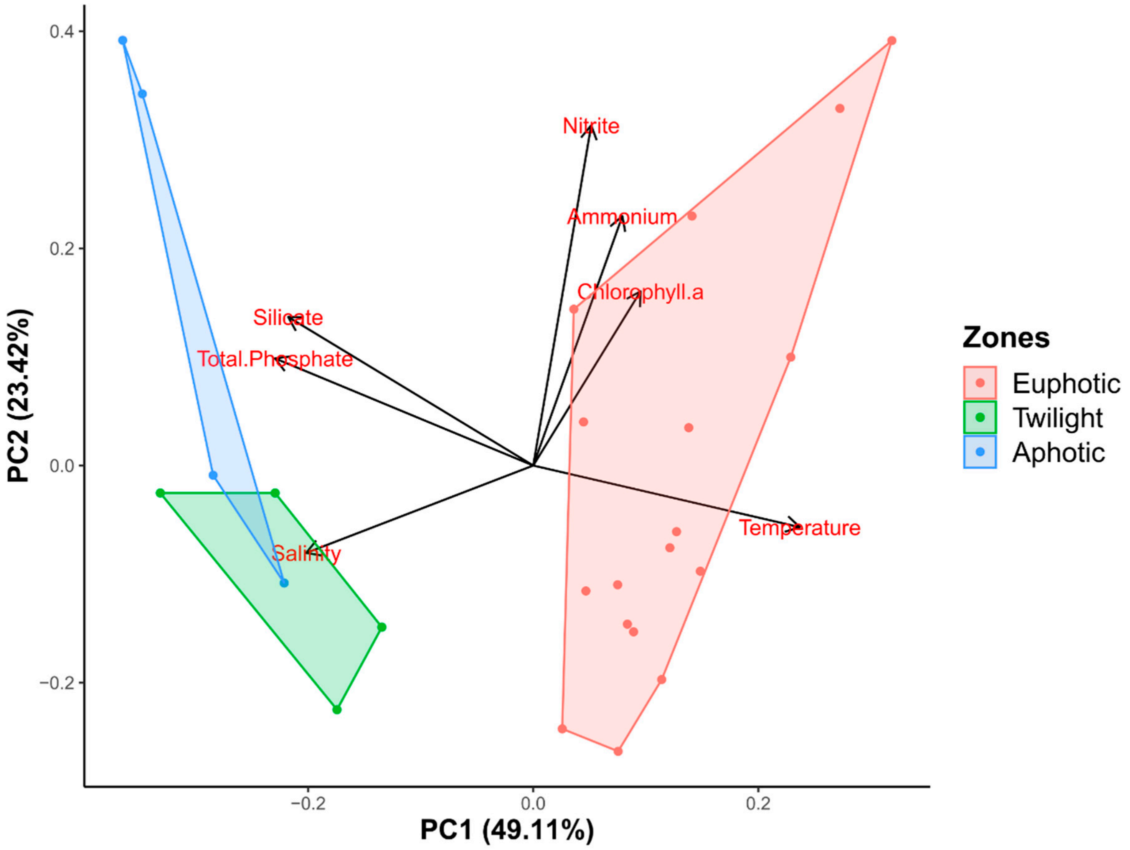

Figure S3. PCA illustrating the variations in the environmental characteristics of various water zones.

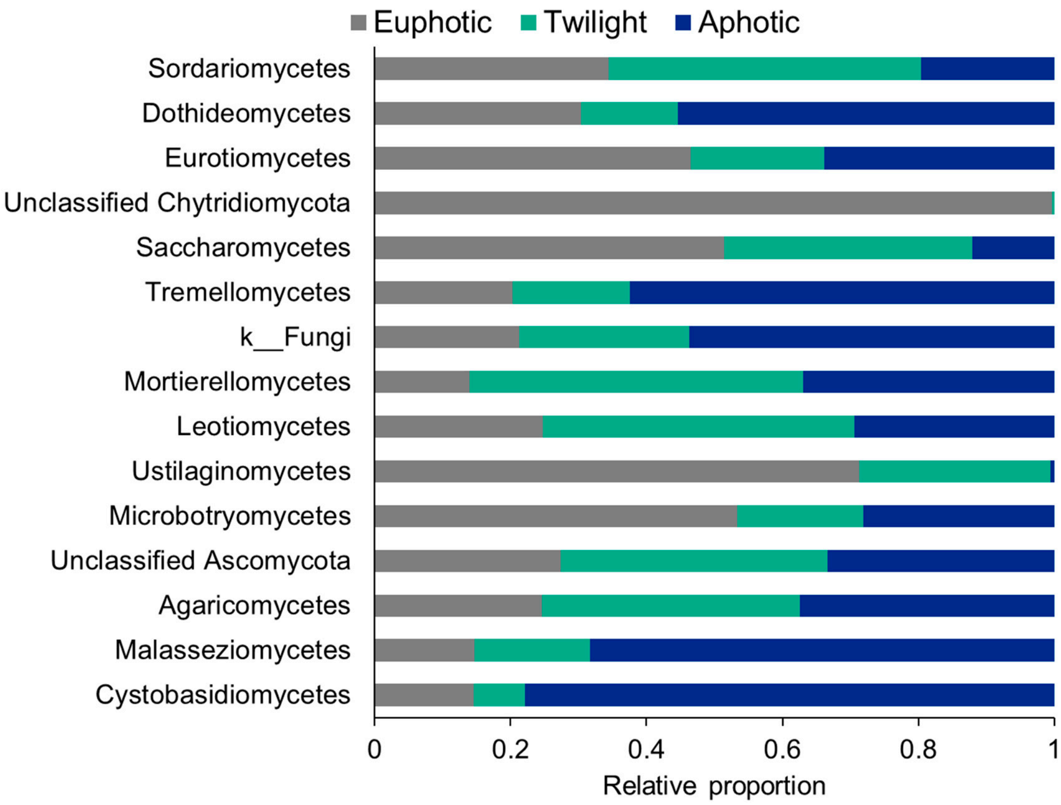

Figure S4. Variations in the taxonomic composition of mycoplankton communities between different water zones at the class level.
